# Actions, Action Sequences and Habits: Evidence That Goal-Directed and Habitual Action Control Are Hierarchically Organized

**DOI:** 10.1371/journal.pcbi.1003364

**Published:** 2013-12-05

**Authors:** Amir Dezfouli, Bernard W. Balleine

**Affiliations:** Brain & Mind Research Institute, University of Sydney, Sydney, New South Wales, Australia; University of Oxford, United Kingdom

## Abstract

Behavioral evidence suggests that instrumental conditioning is governed by two forms of action control: a goal-directed and a habit learning process. Model-based reinforcement learning (RL) has been argued to underlie the goal-directed process; however, the way in which it interacts with habits and the structure of the habitual process has remained unclear. According to a flat architecture, the habitual process corresponds to model-free RL, and its interaction with the goal-directed process is coordinated by an external arbitration mechanism. Alternatively, the interaction between these systems has recently been argued to be hierarchical, such that the formation of action sequences underlies habit learning and a goal-directed process selects between goal-directed actions and habitual sequences of actions to reach the goal. Here we used a two-stage decision-making task to test predictions from these accounts. The hierarchical account predicts that, because they are tied to each other as an action sequence, selecting a habitual action in the first stage will be followed by a habitual action in the second stage, whereas the flat account predicts that the statuses of the first and second stage actions are independent of each other. We found, based on subjects' choices and reaction times, that human subjects combined single actions to build action sequences and that the formation of such action sequences was sufficient to explain habitual actions. Furthermore, based on Bayesian model comparison, a family of hierarchical RL models, assuming a hierarchical interaction between habit and goal-directed processes, provided a better fit of the subjects' behavior than a family of flat models. Although these findings do not rule out all possible model-free accounts of instrumental conditioning, they do show such accounts are not necessary to explain habitual actions and provide a new basis for understanding how goal-directed and habitual action control interact.

## Author Summary

In order to make choices that lead to desirable outcomes, individuals tend to deliberate over the consequences of various alternatives. This goal-directed deliberation is, however, slow and cognitively demanding. As a consequence, under appropriate conditions decision-making can become habitual and automatic. The nature of these habitual actions, how they are learned, expressed, and interact with the goal-directed process is not clearly understood. Here we report that (1) habits interact with the goal-directed process in a hierarchical manner (i.e., the goal-directed system selects a goal, and then determines which habit should be executed to reach that goal), and (2) habits are learned sequences of actions that, once triggered by the goal-directed process, can be expressed quickly and in an efficient manner. The findings provide critical new experimental and computational information on the nature of habits and how they interact with the goal-directed decision-making.

## Introduction

There is now considerable evidence from studies of instrumental conditioning in rats and humans that the performance of reward-related actions reflects the involvement of two learning processes, one controlling the acquisition of goal-directed actions and the other of habits [1]–[4]. This evidence suggests that goal-directed decision-making involves deliberating over the consequences of alternative actions in order to predict their outcomes after which action selection is guided by the value of the predicted outcome of each action. In this respect, action evaluation relies on the representation of contingencies between actions and outcomes as well as the value of the outcomes, which in sum constitute a *model of the environment*. In contrast, habitual actions reflect the tendency of individuals to repeat behaviors that have led to desirable outcomes in the past and respect neither their causal relationship to, nor the value of their consequences. As such, they are not guided by a model of the environment, and are relatively inflexible in the face of environmental changes [5]–[7].

Although these features of goal-directed and habitual action are reasonably well accepted, the structure of habitual control, and the way in which it interacts with the goal-directed process in exerting that control, is not well understood. Two types of architecture have been proposed: a hierarchical architecture and a flat architecture. We have recently described a version of the hierarchical structure in the context of advancing a new theory of habits [8]. Although habits are usually described as single step actions, their tendency to combine or chunk with other actions [9]–[15] and their insensitivity to changes in the value of, and the causal relationship to, their consequences [2], [16] suggests that they may best be viewed as action sequences [8]. On this view habit sequences are represented independently of the individual actions and outcomes embedded in them such that the decision-maker treats the whole sequence of actions as a single response unit. As a consequence, the evaluation of action sequences is divorced from offline environmental changes in individual action-outcome contingencies or the value of outcomes inside the sequence boundaries and, as they are no longer guided by the model of the environment [8], are executed irrespective of the outcome of each individual action [12], [17]; i.e., the actions run off in an order predetermined by the sequence, without requiring immediate feedback.

On this hierarchical view, such action sequences are utilized by a global goal-directed system in order to efficiently reach its goals. This is achieved by learning the contingencies between action sequences and goals and assessing at each decision point whether there is a habit that can achieve that goal. If there is, it executes that habit after which control returns to the goal-directed system. In essence, the goal-directed system functions at a higher level and selects which habit should be executed whereas the role of habits is limited to the efficient implementation of the decisions made by the goal-directed process [8], [18] (see [19] for a review of other schemas).

**Figure 1:**
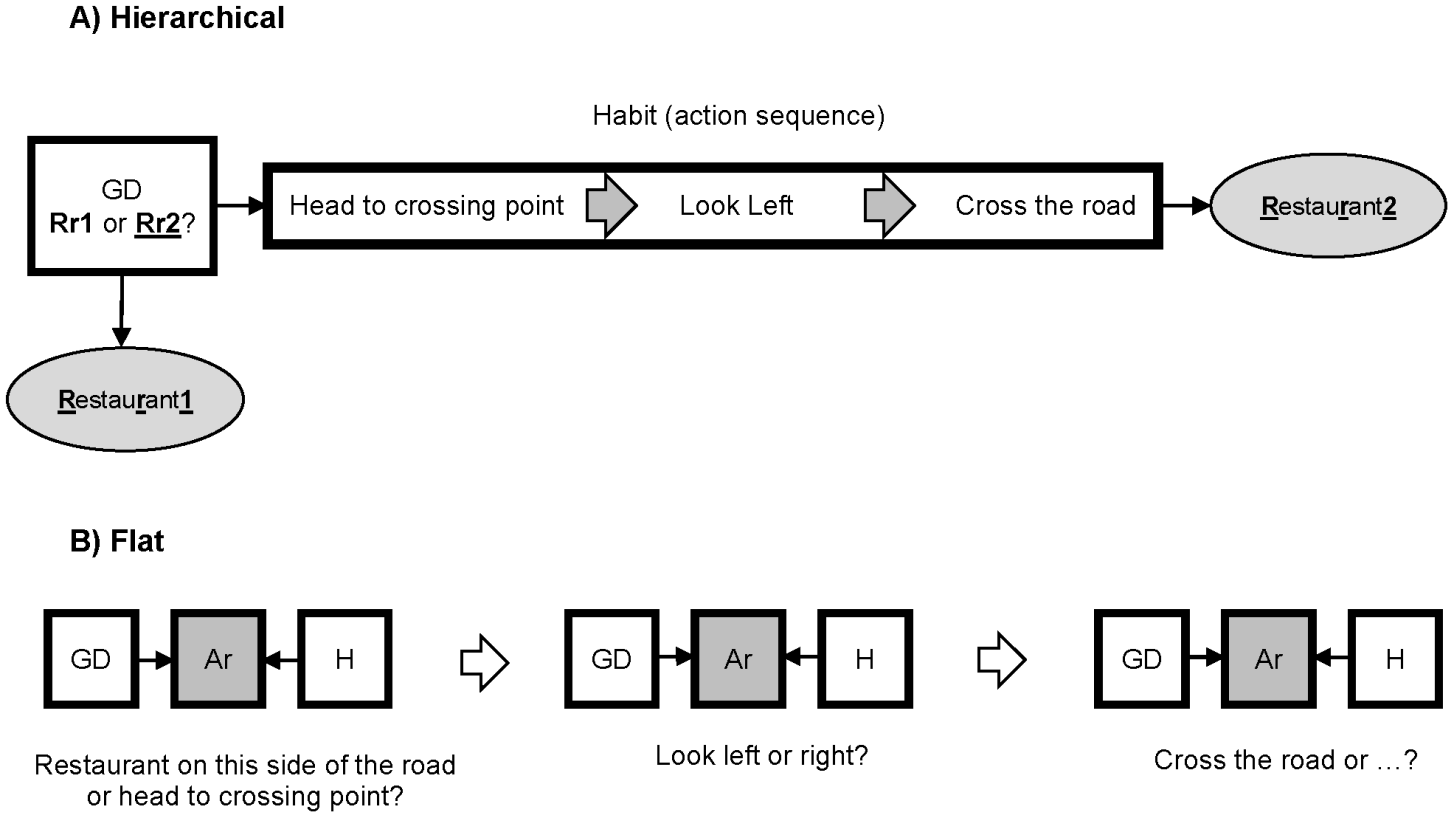
An example illustrating the difference between the hierarchical and flat organizations. (A) Hierarchical interaction. The goal-directed system (GD) selects goals and decides whether to go to a restaurant on this side of the road (Rr1) or on the other side of the road (Rr2). If it chooses to go to the restaurant on the other side of the road, then it triggers the habit of crossing the road and control transfers to the habitual process. After execution of the habit finishes, control returns to the goal-directed system. (B) Flat interaction. At each decision point, the arbitration mechanism (Ar) decides whether the next action should be controlled by the goal-directed system or the habitual system (H).

Assume, for example, you are deciding whether to go to a restaurant on this side of the road or on the other side of the road ( Figure 1A). The goal-directed system evaluates both options, and decides to go to the restaurant across the road. It thus triggers a ‘crossing the road’ habit, and transfers the control to the habitual system. The habit is an action sequence composed of several individual actions: (1) head to the crossing point, (2) look left, and (3) cross the road. Individual actions are executed one after another, and after they finish, the control transfers back to the goal-directed system to make the next decision such as, for example, choosing from the menu in the restaurant.

In contrast to the hierarchical architecture, the flat architecture treats habits as single step actions rather than action sequences (e.g. [5]). At each step, an arbitration mechanism decides whether the next action should be controlled by the goal-directed system or the habitual system. In the context of the above example, at the beginning the arbitration mechanism selects one of the systems to decide whether to go to the restaurant on this side of the road or to the crossing point. Again, at the crossing point, the arbitration mechanism selects one of the systems to decide whether to look left, or right, and similarly at each future step the arbitration mechanism selects one of the systems to control behavior (Figure 1B). It should be clear, therefore, that, in the flat approach, both systems are at the same level and action evaluation happens in both processes; both systems evaluate available alternatives, and the arbitration mechanism determines how these two evaluations combine to make the final decision.

From the flat perspective, another difference between goal-directed and habitual processes lies in how they evaluate actions. The goal-directed process obeys the same principles sketched earlier: learning the model of the environment, and making predictions based on that model (*model-based* evaluation). In contrast, the habitual system is *model-free* and evaluates actions based on their ‘cached’ reward history without searching through the action-outcome contingencies [5], [7], [20]–[22].

More recently, Daw et al [23] have exploited the difference between model-free and model-based evaluation to investigate the interaction of goal-directed and habit processes in a flat structure reasoning that, because model-free evaluation is retrospective, chaining predictions backward across previous trials, and model-based evaluation is prospective, directly assessing available future possibilities, it is possible to distinguish the two using a sequential, multistage choice task. In this task subjects first make a binary choice (the first stage) then transition to the second stage in which they make a second choice to earn a reward. The best choice at the second stage varies depending on the first choice and, to maintain a constant trade-off between habitual and goal-directed systems, the reward probabilities in the second stage are continually varied. By examining first stage choices, Daw et al [23] were able to find evidence of mixed goal-directed and habitual predictions.

Here we show that first stage habitual actions, explained by the model-free evaluation in previous work, can also be explained by assuming that first stage actions chunk with second stage actions, reducing the source of habitual actions to the formation of action sequences. Based on this finding we next examined specific predictions of each account. With regard to the two-stage task, the flat account predicts that feedback received after the execution of an action will affect subsequent decisions and, therefore, that arbitration between goal-directed and habit controllers will recur anew at each stage. As a consequence, action-control at each stage of the task should be independently established; in particular it should be noted that action control in stage two should not depend on stage one. In contrast, because our hierarchical account treats habits as action sequences, and because the execution of habits is open-loop, it predicts that, during the execution of a habit, actions will be executed one after another without considering feedback from the environment during the sequence and, therefore, that, when habitual, the action taken at stage 2 is already determined when starting the habit sequence at stage 1. We made two further predictions from the hierarchical account: first, because of their relative freedom from feedback, action sequences should be elicited more quickly than single actions [24] predicting that, when habitual, reaction times between stage 1 and stage 2 actions will be faster than when non-habitual. Second, and based on these predictions, we anticipated that the hierarchical model would better fit the performance of subjects working on this two-stage task than the flat model.

## Results

**Figure 2:**
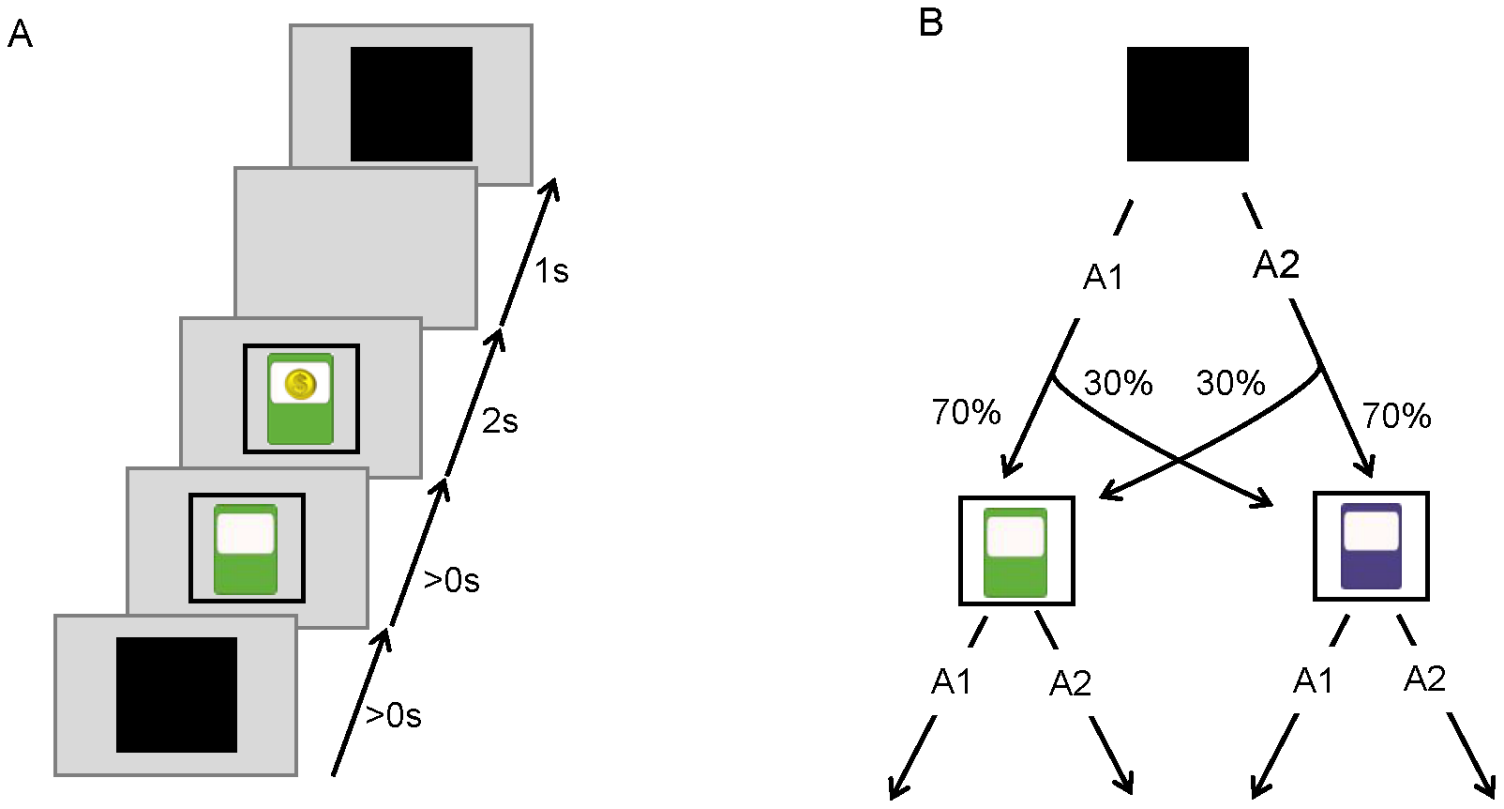
Task description. (A) Illustration of the timeline of events within a trial. Initially a black screen is presented, and the subject can choose between pressing A1, or A2 (first stage choice). After a key is pressed, one of the slot machines is presented, and the subject can again choose between pressing A1, and A2 (second stage choice). Choices at the second stage are reinforced by monetary reward. (B) Structure of the task. One of the key presses commonly leads to one of slot machines (70% of the time), and the other key commonly leads to the other slot machine. Choices at the second stage are reinforced either by a high probability (0.7) or a low probability (0.2). With a small probability (1/7), the rewarding probability of each key changes randomly to either the high or low probability.

Fifteen subjects completed a two-stage decision-making task (Figure 2), in which each trial started with a choice between two key presses (first stage actions; A1 vs. A2). Each key press resulted in the appearance of either of two slot machines (denoted by S1 and S2 and distinguished by their colors) in a probabilistic manner. Next, at the slot machines, subjects again chose between two key presses (second stage actions; A1 versus A2), and, as a result, received an outcome; i.e., either a monetary reward or a neutral outcome. At the first stage, A1 most commonly led to S1, and A2 to S2 (common transitions; 70% of the time). In a minority of trials, A1 led to S2, and A2 to S1 (rare transitions; 30% of the time). This relationship was kept fixed throughout the test. Each of the second stage responses at the slot machines earned a reward either at a high probability (0.7) or a low probability (0.2). In order to ensure the subjects kept searching for the best keys and slot machines during the test, at each trial, with a small probability (1∶7), the rewarding probability of each key changed randomly to either the high or low probability. Each participant completed 270 trials.

### Goal-directed and habitual performance on the two-stage task

In the analysis, we first sought to establish whether decision-making in this task is goal-directed, habitual or a mixture of both and, if both, to assess whether goal-directed and habitual control interact according to a flat structure or a hierarchical structure.

**Figure 3:**
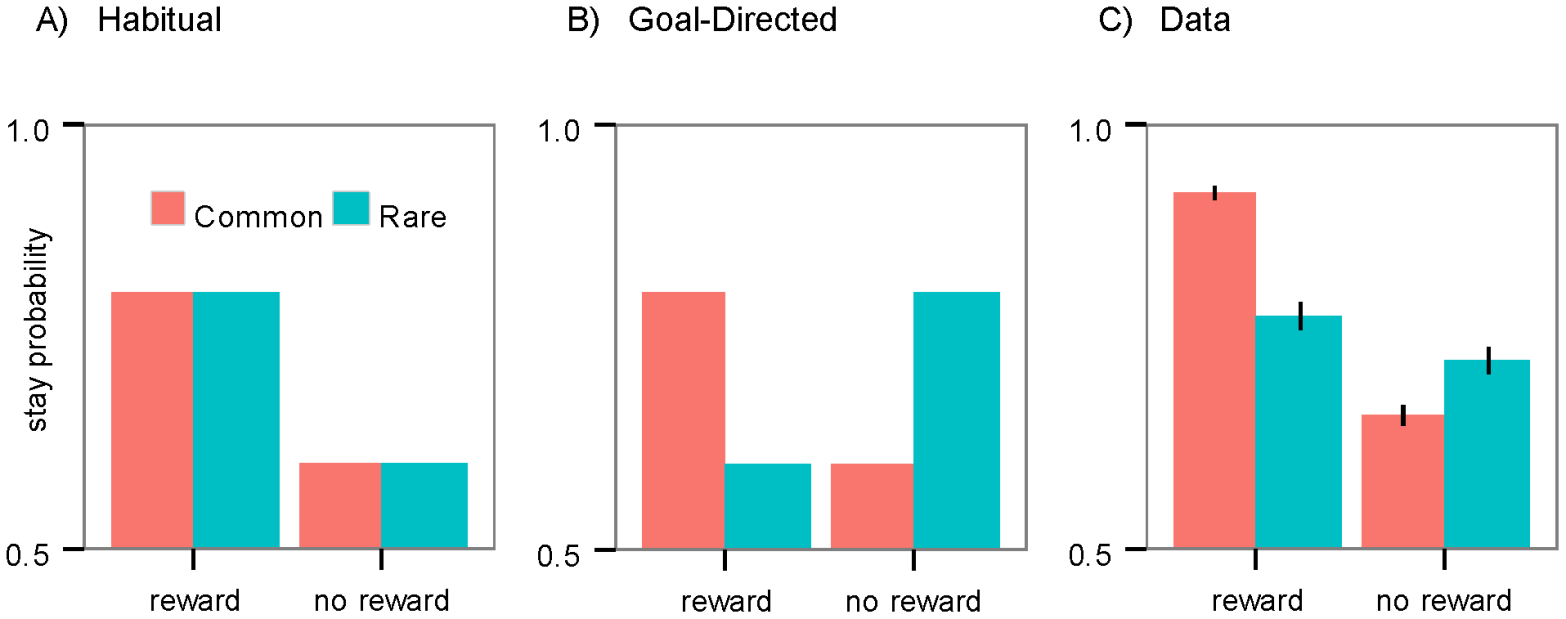
First-stage choices. (A) Modeled habitual action control on the two–stage task: Under habitual control a first stage action that has been eventually reinforced (reward) in the previous trial is more likely to be repeated (higher stay probability), regardless of whether the repeated action commonly leads to the same slot machine (common) or not (rare). (B) Modeled goal-directed action control on the two–stage task: Under goal-directed action control a reinforced action is repeated if it commonly leads to the same slot machine in which reward is received, otherwise the other action is selected. (C) Data from the experiment: Actual stay probabilities averaged over all subjects and trials. When the previous trial was rewarded, stay probability was generally higher (as in habitual control), and was also higher when the previous trial was a common transition (as in goal-directed control). Thus, the responses of the subjects in the experiment were found to be a mixture of both habitual and goal-directed action control. Error bars: 1 SEM.

The first question can be answered by looking at the likelihood of the subjects repeating the same first stage action on each trial based on feedback received on the previous trial [23]. Take for example a trial in which a subject presses A1 and transfers to the S2 slot machine (which is rare result of choosing A1). If the participant presses a button of that slot machine and receives a reward, this implies S2 is probably a good slot machine and, if the decision-making is goal-directed, in the first stage of the next trial the subject should try to reach this S2 slot machine again. It is expected therefore, that the probability that the subject will press A2 will increase because it is this key that (in this example) commonly leads to S2 (cf. Figure 3B). In contrast, if decisions are habitual, subjects should not be guided by contingencies between the responses and slot machines, and should tend to stay on the previously rewarded action, A1 (Figure 3A).

The results are presented in Figure 3C, which shows the probability of repeating the same action computed across all subjects and trials. We analyzed the data using mixed-effects logistic regression analyses by taking all coefficients as random effects across subjects (see Materials & Methods: Behavioral Analysis). Results show that being rewarded in the previous trial increased the chance of staying on the same action, irrespective of whether it was a rare or a common transition (main effect of reward; coefficient estimate = 0.61; SE = 0.09; p<3e-11), which suggests that habits constitute a component of the behavior. On the other hand, this increase was higher if the previous trial was a common transition (and lower after an unrewarded trial), suggesting that subjects also utilized their knowledge about the task structure (reward-transition interaction; coefficient estimate = 0.41; SE = 0.11; p<5e-4). Therefore, the subjects' behavior was a mixture of both goal-directed and habitual actions. Also, as the figure shows, the probability of staying on the same action is generally higher than not staying on it, irrespective of reward and transition type in the previous trial (the intercept term is significantly positive; estimate = 1.52; SE = 0.20; p<10e-14), which reflects a general tendency of animals and humans to repeat previous actions [25]–[27].

**Figure 4:**
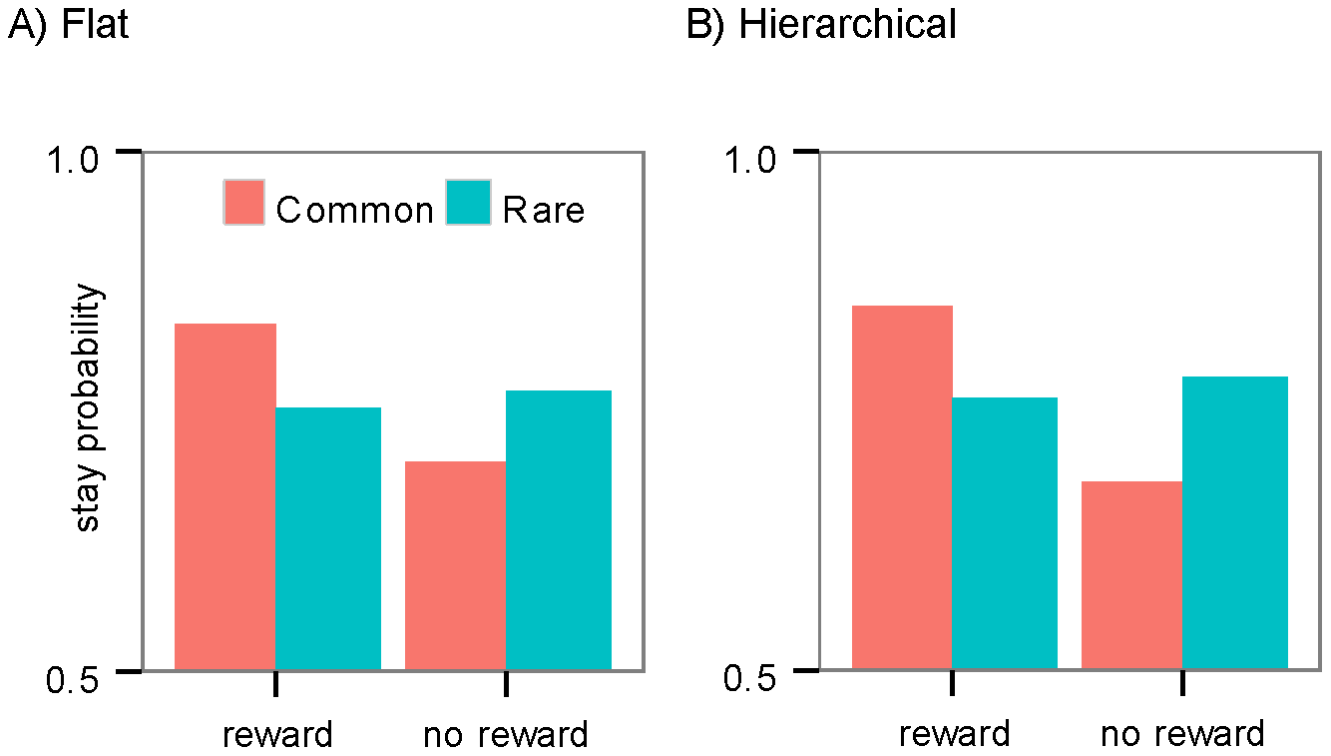
Simulation of the first-stage choices. The probability of staying on the first stage action in simulations of: (A) the flat architecture; and, (B) the hierarchical architecture. Both architectures can model the pattern of data observed in the first stage stay probabilities on the task: i.e., a higher stay probability after being rewarded on the previous trial and an interaction between reward and transition.

In previous studies, a hybrid model of model-free and model-based reinforcement learning (RL) was advanced to explain the behavior of subjects on this task based on the flat structure [23], [28]–[30]. According to this model, action values learned in model-free RL, roughly, reflect the frequency of the action rewarded on previous trials irrespective of the action-outcome contingency (i.e., in the current task, which key generates which slot machine) and, as such, these values underlie the habitual component of the model. These model-free values are then mixed with the values provided by the goal-directed system (modeled by a model-based RL) to produce the final values which guide action selection. As a consequence, and consistent with the above results, we should expect to see a combination of both habitual and goal-directed actions. The prediction from this hybrid model is illustrated in Figure 4A.

A hierarchical structure can, however, also be used to explain these results. For example, assume that a subject presses A1 in the first stage, and A2 in the second stage and receives a reward. As a result, the goal-directed system learns that contingency between the A1A2 action sequence and the reward is increased and so it should be more likely to repeat the action sequence in the next trial, whether or not the reward was received from the S1 or S2 slot machine (i.e., the common or rare transition). As the evaluation and performance of an action sequence is not guided by the task structure (i.e. the key-slot machine association), from this perspective it constitutes the habitual component of the behavior. All actions - either single action (e.g., A1) or action sequences (e.g., A1A2)-, will be subject to the goal-directed action selection process, such that actions with higher values will be selected with a higher probability. As a consequence, this implies that the behavior will be a mixture of habitual (when action sequences are selected) and goal-directed (when single actions are selected) actions and that this mix of actions can be generated without the need for the model-free component or an explicit arbitration mechanism used in the flat structure. This prediction is illustrated in Figure 4B.

### The interaction of goal-directed actions and habit sequences in stage 2 performance

Although both approaches are able to explain the mixture of behavioral control in the first stage, they make different predictions about second stage choices. This is because, if the observed habitual behavior is due to the execution of an action sequence, rather than cached values as the model-free account supposes, then we expect the subject to repeat the whole action sequence in the next trial, not just the first stage action.

Staying on the same first stage action in the next trial after being rewarded implies that this is probably a habitual response and so we expect the subject to repeat the second stage action as well, even if the slot machine is different from the one in the previous trial. In contrast, if the subject switches to the other first stage action, the previous action sequence is not repeated, and thus the second stage action is not expected to be repeated if the subject ends with a different slot machine in the next trial. In order to test this prediction, we looked at the trials that had a different slot machine to the one in their previous trial.

**Figure 5:**
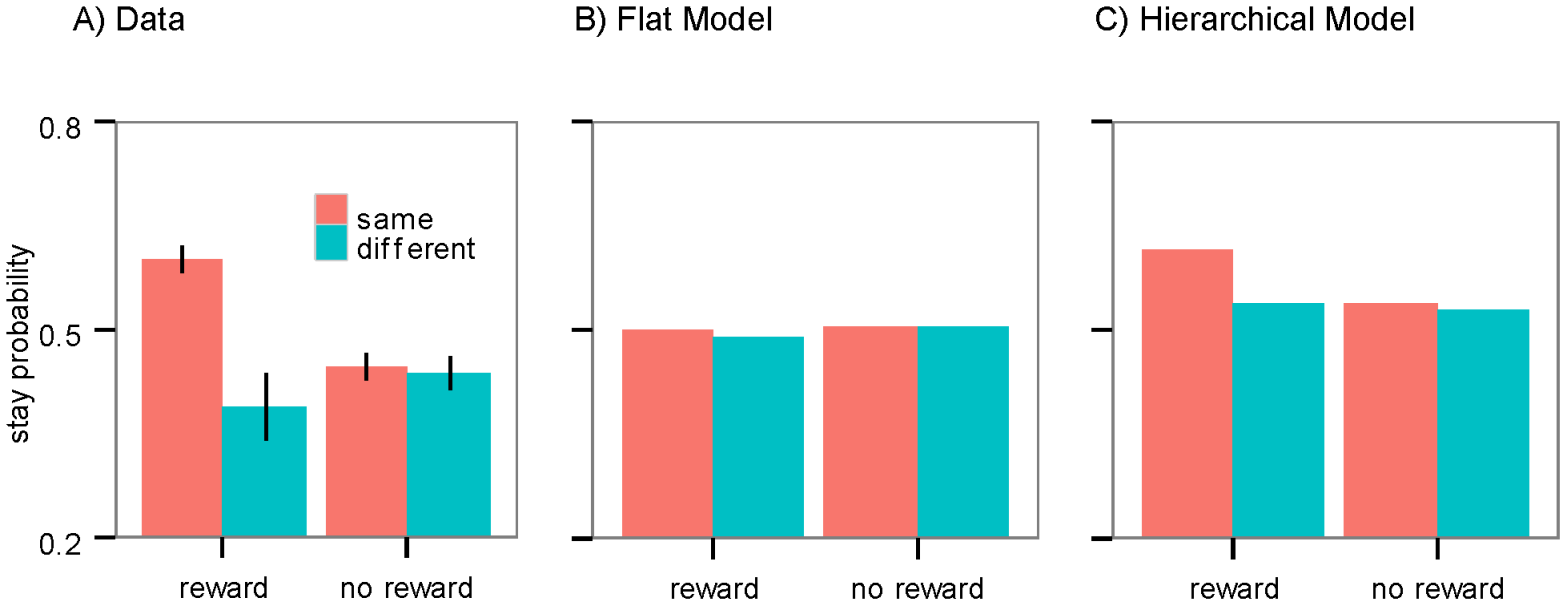
Second-stage choices. The probability of staying on the second stage action on trials for which the slot machine differs from the on one in the previous trial: (A) The observed stay probabilities. When the subjects are rewarded and stay on the same first stage action (same), the probability of staying on the same second stage action is higher. (B) Simulation of the flat architecture. Note that this is not consistent with the pattern in panel (A). (C) Simulation of the hierarchical architecture, which is consistent with the pattern observed in actual stay probabilities. Error bars: 1 SEM.

Figure 5A shows the probability of repeating the same second stage action as a function of whether this action was rewarded on the previous trial and the subject had subsequently taken the same first stage action. Logistic regression conducted on second stage choices using factors of reward, separating rewarded and non-rewarded trials, and action, separating trials on which the first stage action was the same from those on which it differed, found neither an effect of reward (p>0.05), nor of action (p>0.05) but found a significant interaction between these factors (coefficient estimate = 1.02; SE = 0.38; p<0.008), indicating that, during the execution of habitual responses, subjects tended to repeat the second stage action. This interaction remained significant even when we restricted the analysis either to trials after rare transitions (coefficient estimate = 1.33; SE = 0.60; p<0.05) or after common transitions (coefficient estimate = 0.93; SE = 0.38; p<0.05). Importantly, the fact that the effect of the reward was not significant rules out the possibility that the effect was due to the generalization of the values across slot machines.

Simulations of the flat and hierarchical models are presented in Figure 5B and C, respectively. As predicted, the hierarchical structure captures the pattern of the subjects' second stage actions (the interaction between the reward and the same first stage action; p<0.001), whereas the flat structure is not consistent with repeating the same action in the second stage (p>0.05).

Previously, we focused on trials with a different slot machine to the one in the previous trial. This was because, in this condition, flat and hierarchical accounts provide different predictions. When the slot machine is the same, both accounts (flat and hierarchical) predict that being rewarded in the previous trial increases the probability of staying on the same second stage action. In addition to this prediction, the hierarchical account predicts that when the slot machine is the same as the one on the previous trial, this increase should be higher than the increase when the slot machine is different. This is because, when the slot machine is different, staying on the same second stage action is drive by execution of the previous action sequence whereas, when the slot machine is the same, executing either the previous action sequence or a goal-directed decision at the second stage can result in staying on the same second stage action.

As a consequence we looked at the effect of being rewarded in the previous trial, and whether the slot machine was the same as the one in the previous trial, on the probability of staying of the same second stage action (in the trials in which the first stage action was the same as the previous trial).

**Figure 6:**
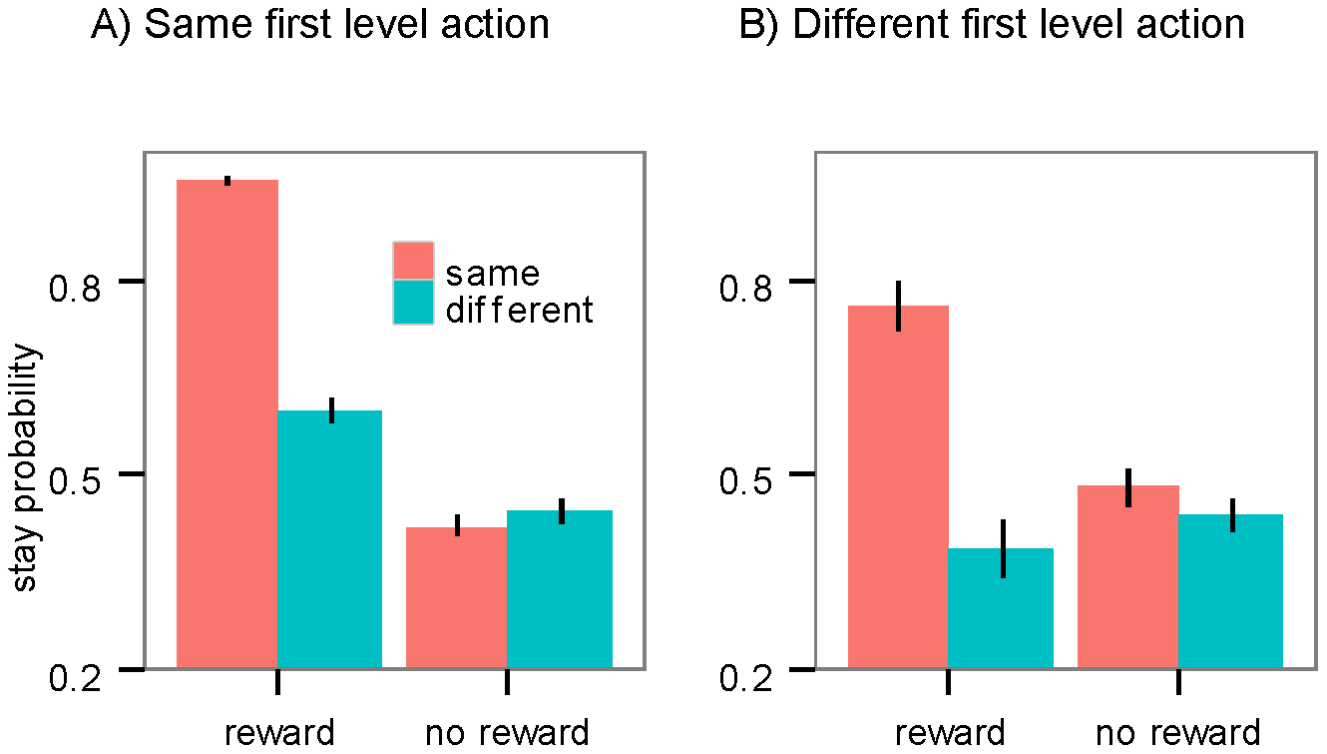
Second-stage choices in a same vs. different state. The probability of staying on the second stage action when the same (A) or different (B) first stage action is taken, as a function of whether the previous trial is rewarded, and whether the second stage state is the same or different from the previous trial. Error bars: 1 SEM.

Figure 6A shows the results. A significant main effect of reward was found (coefficient estimate = 0.69; SE = 0.21; p<0.002) indicating that being rewarded in the previous trial increases the probability of taking the same second stage action, irrespective of whether the slot machine was the same as the previous trial or not, which is consistent with the hierarchical account. In addition, we found a significant interaction between the effect of reward and whether the slot machine being the same (coefficient estimate = 3.46; SE = 0.51; p<3e-11), consistent with the finding that the probability of staying on the second stage action was higher when the second stage action was the same.

Figure 6B shows the probability of staying on the same second stage action when the subject takes a different first stage action. As predicted, because the subject did not execute the previous action sequence, the main effect of reward was not significant (p>0.05) but the interaction between reward and the second stage being the same was significant (coefficient estimate = 1.72; SE = 0.40; p<3e-5) which means that subjects tend to take the same action on the same slot machine after being rewarded, as predicted by both accounts.

### Reaction times during habit execution

In the previous section we showed that if, after being rewarded, the subject repeats the same first stage action, they are probably repeating the previous action sequence and, as such, they tend to repeat the second stage action as well. However, even in the situation in which the subject is executing an action sequence there will be trials on which they might not repeat the same second stage action. In such conditions, we should suppose that either (i) the subject took an exploratory goal-directed action in the first stage, or (ii) the subject started an action sequence but its performance was inhibited and control returned to the evaluation system in the second stage. In both cases, the hierarchical account predicts that reaction times on trials in which the second stage action is not taken should be higher.

**Figure 7:**
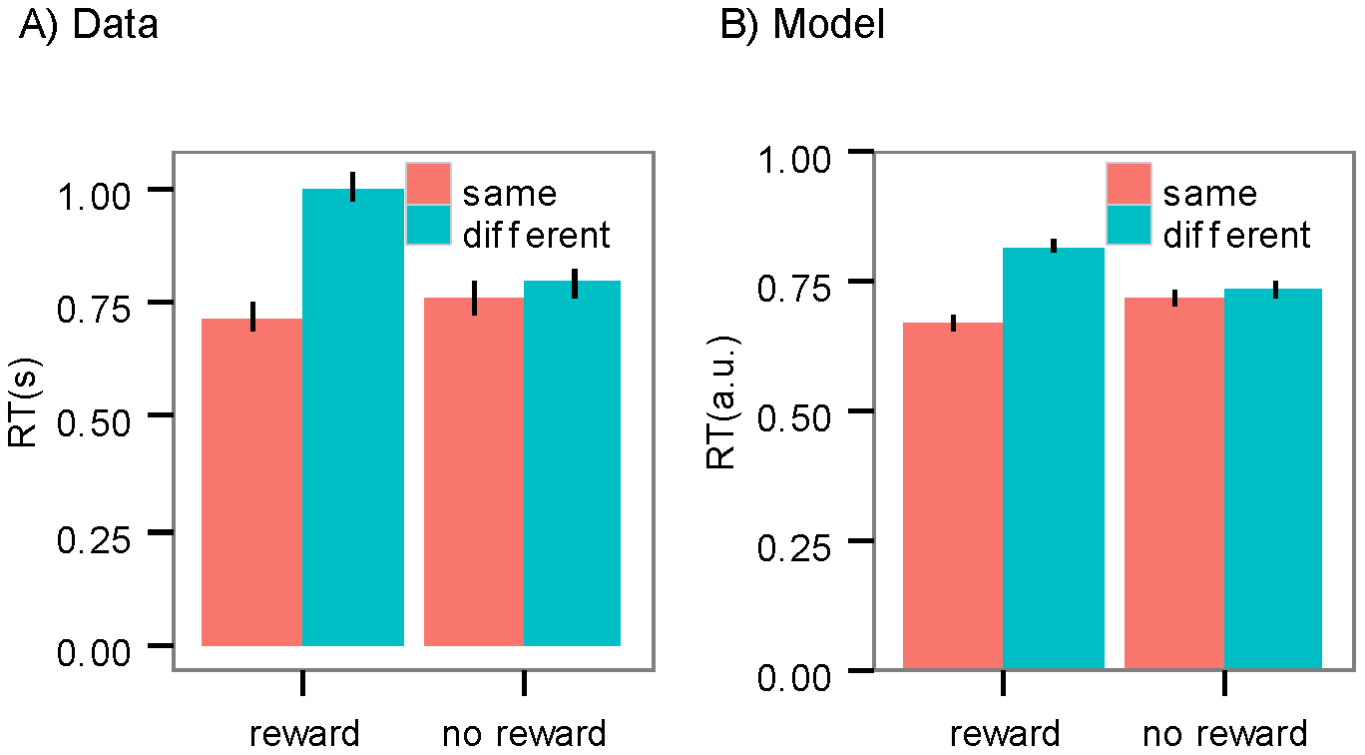
Reaction times in the second-stage. (A) Reaction time (RT) in the second stage action when the same first stage action was taken as a function of whether the same second stage action was taken and whether pervious trial is rewarded (only calculated for trials on which the second stage state was different from the previous trial). (B) Predicted reaction times by the model (a.u. : arbitrary unit). Error bars: 1 SEM.

Figure 7A illustrates these reaction times as a function of whether the previous trial was rewarded and the subject takes the same second stage action (only in trials on which the slot machine is different from that on the previous trial and the subject subsequently takes the same first stage action). If the previous trial is rewarded, reaction times were lower when a subject completes an action sequence than when the second stage action was not executed as a part of a sequence (coefficient estimate = −1.66; SE = 0.45; p<3e-4). Importantly, the effect was not significant when the previous trial was not rewarded (p>0.05), which rules out the possibility that the observed increase in the reaction times was because of the cost of switching to the other second stage action.

We further asked whether the model can predict the reaction times in the second stage. As mentioned above, at the first stage, the goal-directed process more frequently selects actions that have a higher contingency to reward (either single actions, or action sequences). As such, if an action sequence has a high value, it is likely to be selected for execution, and so we expect a low reaction time in the second stage. For example, assume the subject has executed action A1 in the first stage, and A2 in the second stage and the aim is to predict whether A2 has a high or low reaction time. It can be argued, if the value of the A1A2 action sequence is high, that it was probably executed in the first stage, and thus the execution of A2 is part of an action sequence (A1A2) started in the first stage, implying the subject should show a low reaction time. In general we assume that the reaction time in the second stage is inversely related to the value of the action sequence that contains that action (see Material & Methods: Hierarchical sequence based, model based RL). In the case of this example we will have:
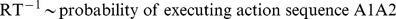
Based on this, we calculated the predicted reaction time of the action taken by the subject in the conditions shown in Figure 7A. The results are shown in Figure 7B. As the figure shows, the predicted reaction times by the model are consistent with the pattern of reaction times observed in the data.

**Figure 8:**
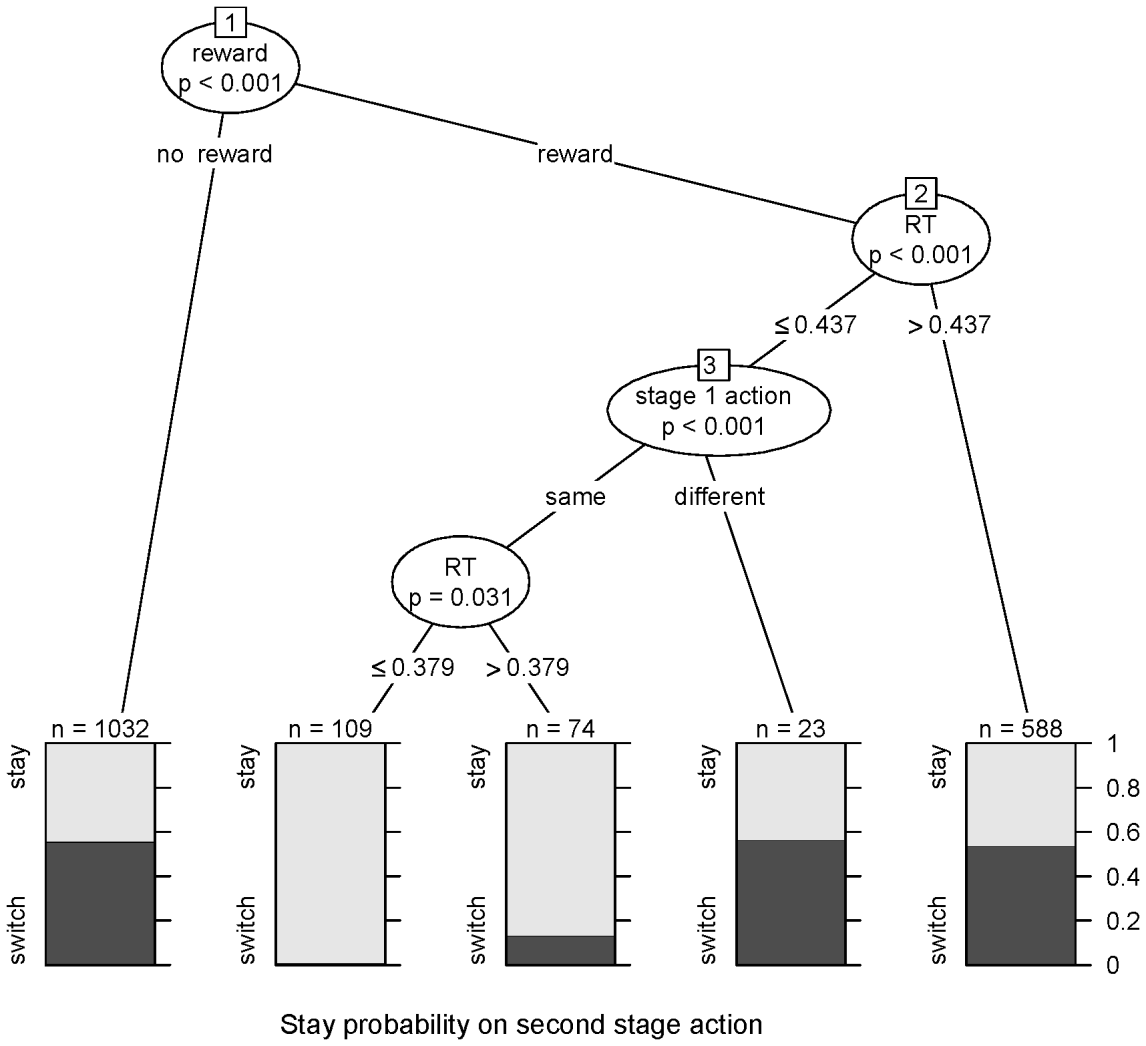
Effect of reward, and reaction times on second-stage choices. Partitioning the probability of staying on the same second stage action (stay: staying on the same second stage action; switch: switching to the other second stage action) as a function of (i) reward on the previous trial (node #1), (ii) whether the same first stage action is taken (stage 1 action; node #3), and (iii) reaction times (RT). ‘n’ represents the number of data points; p-values are calculated using a permutation test.

In general, the above analysis of stage 2 performance and this analysis of reaction times implies that (i) when the previous trial is rewarded, (ii) the same first stage action is taken, and (iii) the reaction time is low, then the subject is most likely performing an action sequence. As a consequence it is expected to repeat the same second stage action, even on a different slot machine to the one in the previous trial. In order to more closely examine this relationship we used conditional inference trees and partitioned second stage actions into whether they involved staying or switching to the other action based on the above three factors (see Materials & Methods: Behavioral Analysis for more details). The results are shown in Figure 8. As the figure shows, when the previous trial was not rewarded (node # 1 ‘no reward’ condition), staying on the same second stage action was independent of either whether the first stage action was repeated or the reaction time was low (p>0.05; permutation test). If the previous trial was rewarded (node # 1 ‘reward’ condition) then, if the reaction time was high (node #2 RT>0.437s) or the reaction time was low but the subject doesn't repeat the first stage action (node #3 ‘different’ condition), then again the second stage action was not repeated. Only when: (i) the previous trial was rewarded, (ii) the subject took the same first stage action, and (iii) their reaction time was low (node #3 ‘same’ condition), did the subject repeat the second stage action, consistent with the prediction of the hierarchical account.

### Behavioral modeling: Bayesian model selection

The results described in the previous sections suggest that a hierarchical structure better characterizes the effect of feedback from the previous trial on performance on the subsequent trial. However, choices are generally guided by the feedback from all previous trials, not just the immediately prior trial. As such, it is still to be established which framework better captures behavior in this more general condition.

We used a Bayesian model selection method to establish which framework produces choices that are the most similar to the subjects' actions. Both flat and hierarchical architectures have different variants with different degrees of freedom. As such, we compared a family of flat models with a family of hierarchical models [31], where each family consists of a complex model, and its nested simpler models. The results (Table 1) show that, given the subjects' data, the hierarchical family is more likely than the flat family to produces choices similar to those made by the subjects. We found that the exceedance probability in favor of the hierarchical family was 0.99 meaning, roughly, that we can be 99% confident that the hierarchical family generated the observed data.

**Table 1:** Model comparison between hierarchical and flat families.

				In Each Family		In Total		
Family	Free Parameters	-log(P(D|M))	p-r^2^	Number Favoring Best Model	Exceedance Probability	Exceedance Probability	Number Favoring Best Model	Exceedance Probability
H	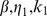	4219.6	0.26	12	0.000	0.004	12	0.993
	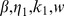	4092.7	0.29	13	0.000	0.003	13	
	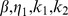	4189.3	0.27	12	0.001	0.005	12	
	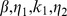	4127.9	0.29	11	0.003	0.013	11	
	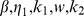	4074.9	0.30	11	0.002	0.011	11	
	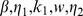	4078.0	0.30	10	0.004	0.011	10	
	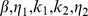	4110.3	0.29	11	0.000	0.004	11	
	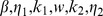	4058.7	0.30	-	0.986*	0.911**	-	
F	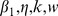	4212.0	0.27	14	0.004	0.002	14	0.006
	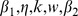	4212.5	0.27	12	0.004	0.004	13	
	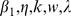	4168.6	0.28	-	0.697*	0.006	12	
	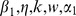	4201.1	0.27	12	0.005	0.002	14	
	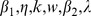	4173.1	0.28	9	0.032	0.006	11	
	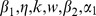	4198.4	0.27	11	0.007	0.003	13	
	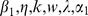	4174.2	0.28	11	0.049	0.002	12	
	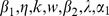	4169.4	0.28	10	0.199	0.005	12	

H: Hierarchical; F: Flat. Shown for each model: negative log model evidence −log(P(D|M)); a pseudo-r statistic (p – r^2^) which is a normalized measure of the degree of variance accounted for in comparison to a model with random choices; the number of subjects favoring the best fitting model based on the model evidence; The exceedance probability which represents the probability that each model (or family) is most likely among alternatives over the population.

* best fitting model in each family.

** best fitting model.

In the hierarchical family, the probabilities of taking actions in the second stage are partially based on the probability of taking an action sequence in the first stage. As these second stage choices are the canonical difference between the two families, we expected that removing the effect of action sequences on the second stage choices would reduce the fit of the hierarchical account to data. Thus we generated a family of hierarchical models similar to Table 1. but with the effect of action sequences on the second stage actions removed, and compared the generated family with the family of hierarchical models presented in Table 1 (see Materials & Methods: Hierarchical model-based, sequence-based RL). Results indicated that the exceedance probability in favor of the family in which the performance of action sequences was reflected in second stage choices was 0.99, confirming that the selection of an action sequence in the first stage increased the probability of taking the second element of the action sequence in the next stage.

Table 1 represents the model comparison results within each family. The parameter estimates for the best fitting model from each family in terms of the exceedance probabilities [32] are presented in Table 2. The best fitting models from each family were simulated in the task conditions to produce Figure 4 and Figure 5.

**Table 2:** Best fitting parameter estimates for each family across subjects.

Hierarchical				Flat			
Parameter	First Quartile	Median	Third Quartile	Parameter	First Quartile	Median	Third Quartile
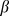	4.24	5.80	6.96	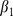	1.64	2.33	3.44
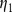	0.82	0.89	0.95	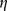	0.65	0.84	0.93
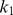	1.25	1.66	2.25	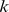	0.84	0.94	1.40
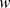	0.25	0.46	0.62	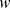	0.40	0.59	0.68
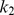	−0.50	0.30	0.70	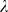	0.69	0.93	0.95
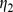	0.14	0.29	0.77	-	-	-	-

## Discussion

Although prior research has suggested that goal-directed and habitual actions should be conceived as single step actions organized according to a flat architecture (e.g. [5], [23]), the results of the current experiment found that: (i) human subjects combined actions together to form action sequences, as revealed by the open-loop execution of sequences of actions and reaction times in the current task, and, therefore, that action sequences constituted a necessary component of behavior; (ii) the use of action sequences by human subjects was sufficient to explain habitual decisions on this task, meaning choices that were not guided by action-outcome contingencies; and, (iii) a goal-directed system assessing both actions and action sequences in a hierarchical manner explained behavior better than a flat model attributing habits to model-free evaluation.

Furthermore, although hierarchical models have had a longstanding role in decision-making [11], [13], [15], [33]–[37], here we provide direct experimental evidence for the role of these models in understanding the operation and interaction of goal-directed and habitual actions. We used a version of the two-stage discrimination task described by Daw et al [23] in which the ambiguity of the first stage predictions by both actions and stimuli was reduced by removing the explicit predictive cues of previous versions. Using this task we found, as previously described, that action selection in the first stage reflected a mixture of goal-directed and habitual strategies. The two accounts diverge with respect to the status of the second stage actions; whereas the flat architecture/single step action perspective predicts that the status of action selection in the second stage should be independent of the first, we found that this was not true; habitual action selection in the first stage predicted continued habitual selection in the second stage as a sequence of actions, a finding predicted by a hierarchical goal-directed/habit sequence account [8]. According to this account, at the top of the hierarchy the goal-directed system evaluates and selects goals and then habits efficiently implement decisions made by the goal-directed system in the form of action sequences. In comparison to the other accounts, which posit a flat interaction between these two systems, we found that the hierarchical account provides more accurate predictions both in terms of the choices of the subjects, and in terms of their reaction times during action selection. When performing according to a habitual sequence of actions, subjects tended to repeat both previously reinforced sequences and to perform these sequences at significantly lower reaction times than when their actions were goal-directed.

### Hierarchical decision-making and the two-stage task

A number of studies have previously investigated the relationship between hierarchical RL and decision-making [7], [19], [38]–[43]. We extended these studies by showing how the formation of action sequences can lead to decisions that are insensitive to (i) the values of the outcomes [8] and (ii) the contingency between specific actions and their outcomes (i.e. the key press–slot machine associations in this study), the two defining characteristics of the habitual behavior.

The other difference between the hierarchical RL model that we used here and previous work is that we assumed that performance of action sequences is insensitive to the feedback received during execution [12], [17], whereas, in general, previous work based on hierarchical RL theory has assumed that action selection is based on the state of the environment [44]–[46]. Within this latter framework, one can posit that habits are hierarchically organized actions but that their performance is sensitive to the feedback received after execution of each individual action. Although this class of models can explain habitual behavior executed in the first stage of the current task, this approach predicts that second stage actions will, ultimately, be similar to those of the flat architecture discussed earlier, which is not consistent with the data observed in this study.

In the hierarchical account advanced here we assumed, based on the previous findings in rodents [18], [47], that, similar to single actions, action sequences are also under goal-directed control. Alternatively, it is possible that the value of any action sequence is learned in a model-free manner (for example using Q-learning) without learning the identity of the particular outcome that it predicts. Our results are silent with respect to this latter assumption; nevertheless, whatever the case, the conclusion that habitual responses in the first stage were due to the execution of an action sequence still holds. One way to study this issue is to add another choice to the end of the task, making it a three stage task, and then asking whether performance of for example A1A2 action sequence is goal-directed or habitual, which can be answered by devaluation of outcome of A1A2, or using the same task structure that we used here to distinguish habitual and goal-directed actions. However, again, if it were found that the selection of the A1A2 sequence was not sensitive to environmental contingencies, or outcome values, this could be due either to the formation of A1A2A3 action sequence (since outcome of A1A2 falls within sequence boundaries [8]), or it could be because action sequences are open to model-free evaluation. Similar to the study here, these accounts can be distinguished by examining whether the subject selects A3 during habitual selection of A1A2 irrespective of the outcome of A1A2 performance. If so, it can be concluded that the observed habitual behavior is due to the formation of an action sequence, not model-free RL. Along the same lines, it is possible to assume that, in the current study, first stage habitual responses were guided by a flat model operating in parallel to the hierarchical model we propose here. Again, although the task results are neutral with respect to this assumption, adding a parallel model increases the model's complexity, is not required to account for the current data, and so it necessity should be motivated by additional behavioral data.

It might also be argued that, although the current predictions apply to the modified two-stage discrimination task used here, they may not apply to previous versions of the task. In previous versions, subjects at each stage chose between two symbols instead of two fixed actions and the symbols moved from side to side at each trial ensuring there was no consistent mapping between the button presses and the symbols. There are two points to make here: First, the fact that specific (e.g. left- or right-hand) actions are degraded in their contingency with the outcome on this version of the task raises the issue of stimulus control; either the stimuli exclusively mediate the predictions of second stage outcomes or the concept of action needs to be made more liberal to the selection of a symbol. The former approach would, of course, render the task Pavlovian, rather than instrumental, and the applicability of model-based control problematic. Second, and relatedly, in order to apply our hierarchical model to the earlier task, we also need to extend the concept of an ‘action’ from pressing a button (as in our task), to selecting a symbol; if this is accepted then, using the logic laid out earlier, the hierarchical goal-directed/habit sequences model can explain the results of the task. In the prior version of the task, symbols in the second stage were different from each other, for example in one of the second stage states subject could choose between symbols ‘C’ and ‘D’, but in the other second stage state, the choice was between symbols ‘E’ and ‘F’. As such, we cannot directly assess the probability of staying on the same second stage action if the subjects end up in a different second stage state. Nevertheless, the hierarchical theory predicts that if the subject selects same first stage action, and ends up with the same second stage state and selects the same second stage action, then the reaction time will be faster than when they end up with in a different second stage state.

### Deviations from prediction and the interpretation of the two-stage task

Predictions of both models (flat and hierarchical) were found to deviate from the behavior of the subjects in two cases. In the first case, if, after being rewarded, the subject switches to the other action then both accounts predict that the probability of staying on the second stage action should be on average 0.5 (Figure 5B,C). However, in the actual data it is below 0.5 (Figure 5A). In the second case, both accounts predict that the difference between stay probability in common and rare transitions should be equal in both the reward and no-reward conditions (Figure 4A, B), however, as Figure 3C shows, the difference is larger in the reward condition. It is possible to capture these two deviations by adding more free parameters to the models; however, since the deviations exist for both the flat and hierarchical families and so do not affect the comparison between them, we didn't add further parameters to account for these two deviations.

As in previous work, we interpreted the interaction between being rewarded and the type of transition in the previous trial (rare or common) as the evidence for goal-directed behavior. It should, however, be noted that, if there is a strong initial bias in total possible reward for one action vs. the other at the first-stage, and reward transitions are slow, then it is possible to observe an interaction between reward and transition type without engaging a goal-directed system. As a consequence of the higher overall probability of reward for taking, say, action ‘A1’ in the first stage, the subject can establish that action has a higher value (without relying on the task structure) and so will take that action, i.e. ‘A1’, more frequently than the other, i.e. action ‘A2’, which means that the probability of staying on action ‘A1’ will be higher than action ‘A2’ in general. At the same time, because action ‘A1’ is better than the other action, most of the rewarded common transitions and unrewarded rare transitions result from taking action ‘A1’. Likewise, most of the unrewarded common transitions and rewarded rare transitions will be the result of taking action ‘A2’. This fact, and the fact that stay probability on action ‘A1’ is generally higher, will produce a reward-transition interaction, without having a goal-directed system, at least in the period that action ‘A1’ is better than the other action. This bias is proportional to how fast the bias in first stage values changes and cannot account for the current data. It should also be noted that, as the comparison between the flat and hierarchical model families was based on model fit, those results don't suffer from this problem.

### Inhibitory interactions between goal-directed and habitual control

Although, on the hierarchical goal-directed/habit sequence model advanced here, habits are integrated with the goal-directed process to reach the goals selected by this latter system, competition can also occur between these two systems when the further execution of an ongoing habit sequence is found to be inappropriate by the goal-directed system and it attempts to take back control. This type of competition resembles the situation in an inhibitory control task, such as the stop-signal task, in which subjects must respond quickly when a ‘go’ signal appears but must stop the action if a stop-signal appears [48]. In the context of our task, seeing a slot machine in the second-stage is the ‘go’ signal, which causes the execution of the next action in the sequence. The stop signal comes from the goal-directed system when the pending response is identified as inappropriate. Consistent with this conception in conditions in which sequence performance is inhibited, reaction times are slower. In the stop-signal task, subjects are typically able to inhibit their responses when the stop signal is temporally close to the ‘go’ signal. Although the stop signal task is more global in terms of response inhibition, whereas in the current task the inhibition is specific to one as opposed to an alternative action, this implies that the ability of the goal-directed system to override habits depends on how fast it calculates the correct action: the faster it calculates, the higher the chance of taking control back before action execution.

### Habit sequences vs. stimulus-response habits

It is also interesting to consider the relationship between habit sequences and stimulus-response (S-R) theories of habit learning. The S-R theory of habit learning maintains that habits are responses that are elicited by antecedent stimuli rather than their consequences [49], [50]. Such S-R theories maintain that stimuli trigger their associated behavioral responses due to an association between the stimulus and the response. According to the habit sequence theory, however, the stimulus instead signals that the next action in the sequence should be executed; i.e., in the context of our task, seeing a slot machine signals that it is time for the next action to be executed. Although the next action to be executed is determined by the sequence, the response is still stimulus-bound to some extent and is elicited only when the next expected stimulus is encountered. Nevertheless, these two theories provide different predictions. For example, S-R theory predicts that, in the presence of the appropriate stimulus the response will be performed, irrespective of whether that stimulus was encountered as part of the habit sequence or not. In contrast, habit sequence theory predicts that the individual will respond to the stimulus only when the appropriate habit sequence has already been launched by the goal-directed system.

### Action sequence formation, error signals and dopamine

In the two-stage task that we used in this study, there are few possible action sequences, and so it is easy for the subject to enumerate all of them during decision making. However, in general, the number of action sequences grows exponentially with number of individual actions, and, as such, it will rapidly become impractical to consider all of them at the choice point. As a consequence, the decision-maker needs to discover ‘useful’ action sequences, and to limit consideration to those for action selection rather than all the possible action sequences. In the context of the hierarchical RL literature, this problem is known as ‘option discovery’ and various methods has been proposed to address it (see [19] for a review). In particular, we have previously shown how action sequences can be formed using a reward prediction error signal [8], which has the benefit of forging a bridge between habit sequence formation, and reward prediction error which has been shown to be coded by the phasic activity of dopamine neurons in midbrain [51], [52].

The flat architecture also utilizes reward prediction error, but for the learning of S-R associations instead of action sequences [5]. Here one critical difference lies in the fact that the hierarchical architecture maintains that the reward prediction error is not computed at the second stage when actions are executed habitually in contrast to the flat architecture according to which reward prediction errors are computed in all conditions.

## Materials and Methods

### Participants and behavioral task

Fifteen English speaking subjects (seven females; eight males; mean age 23.8 years [SD 4.3]) completed a two-stage decision-making task. After a description of the study, written consent was obtained. This study was approved by the Sydney University Ethics Committee.

Each subject completed 270 trials, with a break after the first 120 trials (Figure 2). Each trial started with the presentation of a black square and subjects could choose between pressing either ‘Z’ (using left hand) or ‘/’ (using right hand). After pressing the key, a slot machine appeared on the screen, and the subject could make the next response, which would result in either a monetary reward or no reward. The outcome was shown for two seconds and after that an inter trial interval started and lasted for one second, after which the next trial began.

The probability of earning money at each choice was randomly set to either 0.2 or 0.7 at the beginning of the session, and in each trial, with the chance of 1/7, they were again randomly set to 0.2 or 0.7. This later step was to encourage searching for the best keys throughout the session.

Subjects were instructed that the chance of reaching each slot machine by pressing each key will not change throughout the task, but the goodness of the keys in terms of leading to rewards will change over time.

If a first stage action is the best action (the maximum probability of receiving reward on the keys of the slot machine that it commonly leads to is greater than the other action), and slot machines reset in the next trial, the probability that the action remains the best action is 3/16. Based on this, and given that probability of resetting is 1/7, the average number of trials for which a first stage action remains the best action is as follows:
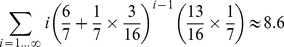
(1)The fact that a first stage action remains the best for a few numbers of trials ensures that reward-transition interaction does not emerge as the result of developing bias toward the best action.

### Behavioral analysis

For all the analyses, we used *R* [53], and the *R* package *lme4* [54]. 

In the analysis presented in the section headed ‘Goal-directed and habitual performance on the two-stage task’, we used mixed-effects logistic regression in which whether the previous first stage action is repeated was a dependent variable, and the transition type (rare or common), and reward received in the previous trial were explanatory variables. We treated all the explanatory variables as random effects.

In the analysis in the section headed ‘The interaction of goal-directed actions and habit sequences in stage 2 performance’, staying or switching to the other second stage action is the dependent variable, and the reward received in the previous trial and staying on the first stage action were the explanatory variables. Only trials in which the second stage states were different from previous trials were included in this analysis. All the explanatory variables were used as random effects. In the second analysis of this section, staying on the same second stage action is dependent variable, and whether second stage state is the same, and whether previous trial was rewarded, are explanatory variables, and also random effects. Only trials in which first stage action is the same as the previous trail were included in this analysis. The third analysis is similar to the third one, except that trials in which first stage action is not the same as the previous trial are included in the analysis.

For analysis of the model behavior in the section headed ‘The interaction of goal-directed actions and habit sequences in stage 2 performance’, each model was simulated 3000 trials in the task with the best fitting parameters of each individual (see the section headed ‘Computational Modeling’ below for more information). Then we analyzed data using linear mixed-effects regression in which the probability of selecting the same second stage action by the model was taken as the dependent variable, and the reward received in the previous trial and staying on the first stage actions were explanatory variables. The intercept was treated as the random effect, and reported p-values are MCMC-estimated using *R* package *LanguageR* [55].

In the analysis in the first part of the section headed ‘Reaction times during habit execution’, staying on the same second stage action was a dependent variable, and the reaction time was an explanatory and random effect. Only trials in which the previous trial was rewarded (first analysis) or not rewarded (second analysis), the first stage action was repeated, and the second stage state was not the same, were included in this analysis.

In the second analysis of this section, we applied a recursive partitioning method by taking (i) whether the previous trial is rewarded, (ii) whether the same first stage action is being taken, and (iii) reaction time as covariates, and staying on the same second stage action as response. We used *R* package *‘party’* [56] for the analysis which employs conditional inference trees for recursive partitioning. In short, the partitioning method works as follows: at each stage of partitioning the algorithm performs a significance test on independence between any of covariates and the response using permutation tests. If the hypothesis is rejected (in the current analysis p-value less than 0.05), it selects the covariate which has strongest association with the response, and performs a split on that covariate.

### Computational modeling

#### Simulation environment

We assumed that the environment has five states; the initial state denoted by 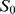, (the black screen in Figure 1), slot machine states denoted by 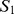 and 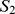, the reward state denoted by 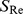 and no-reward state denoted by 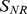.

#### Model-based, model-free RL hybrid

For modeling the flat interaction, a family of hybrid models similar to the previous works was used [23], [28]–[30]. A model-based RL [57] model was used for modeling goal-directed behavior; and a Q-learning model [58] was used to model the habitual behavior. We assumed that actions 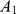 and 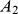 are available in states 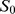, 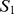 and 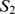.

*Model-based RL*- we denote the transition function with 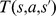 which is the probability of reaching state 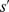 after executing action 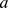 in state 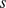. We assume that the transition function at the first stage is fixed (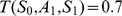 and 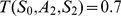) and it will not change during learning. For other states, after executing action 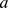 in state 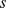 and reaching state 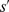, the transition function updates as follows:
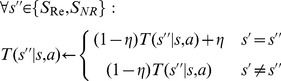
(2)Where 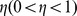 is the update rate of the state-action-state transitions.

We assumed that the reward at state 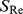 is one (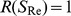), and zero in all other states. Based on this, the goal-directed value of taking action 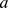 in state 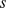 is as follows:
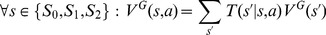
(3)Where:
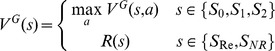
(4)


*Model-free RL*- After taking action 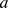 in state 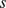, and reaching state 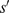, model-free values update as follows:
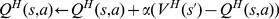
(5)Where 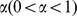 is the learning rate, which can be different in the first stage and second stage actions. For the first stage actions (actions executed in 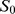), 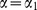, and for the second stage actions 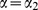. Also
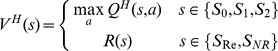
(6)In the trials in which the best action is executed in 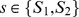 the habitual value of the action executed in state 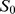 also updates according to the outcome. If 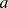 was to be the action which was taken in 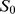, 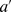 the action taken in 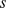, and 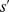 the state visited after executing 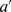, values update as follows:
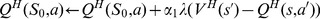
(7)Where 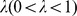 is the reinforcement eligibility parameter, and determines how the first stage action values are affected by receiving the outcome after executing the second stage actions.

Final values are then computed by combining the values provided by the habitual and goal-directed processes:
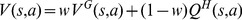
(8)Were 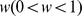 determines the relative contribution of habitual and goal-directed values into the final values.

Finally, the probability of selecting action 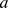 in state 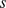 will be determined according to the soft-max rule:
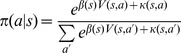
(9)Where 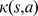 is the action preservation parameter and captures the general tendency of taking the same action as the previous trial [25], [27]. Assuming 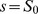 and *a* being the action taken in the previous trial in the 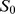 state, then 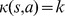, otherwise it will be zero. The 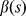 parameter controls the rate of exploration, and 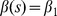 if 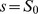 and 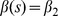 if 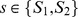.

In the most general form, all the free parameters are included in the model: 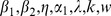 (we assumed that 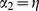). We generated eight simpler models by setting 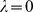, 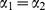, and 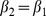.

#### Hierarchical model-based, sequence-based RL

Implementation of the hierarchical structure is similar to hierarchical RL [44]–[46], with action sequences (A1A1, A1A2, etc) as options [46]. We assumed in state 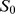, actions 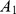, 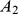, 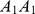, 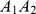, 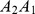, and 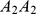 are available. In states 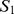 and 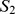, actions 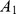 and 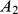 are available. After reaching a terminal state (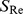 or 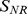), transition functions of both the action sequence, and the single action that led to that state update according to equation (2). In the case of single actions, the transition function will be updated by the 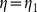 update rate, and in the case of action sequences, the transition function will be updated by the 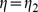 update rate. Based on the learned transition function, value of action *a* in state *s* is calculated by the goal-directed system using equation (3).

The probability of selecting each action will be as follows:
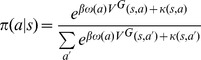
(10)Where 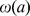 determines the relative preference for single actions instead of executing action sequences. If action *a* is a single action 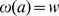, and if action *a* is an action sequence, 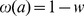. As before, 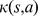 captures action perseveration. We assumed that 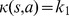 if action *a* is a single action, and 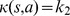 if action 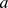 is an action sequence. 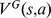 is calculated using equation (3).

For calculating the probability of selecting actions in the second stage, given the first choice of the subject, we need to know whether that action is a part of an action sequence selected earlier, or is it under goal-directed control. Assume we know action 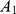 has been executed in state 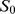 by the subject, the probability of this action being due to performing the 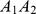 action sequence is:
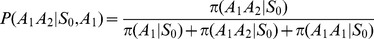
(11)Similarly, the probability of observing 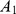 due to selecting the single action 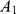 is:
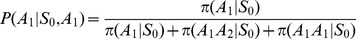
(12)Based on this, the probability that the model assigns to action *a* in state 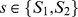, given that action 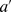 is being observed in 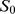 is:
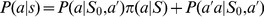
(13)Where 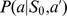 and 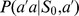 are calculated using equations (11) and (12) respectively. In the most general form, all the free parameters are included in the model: 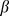, 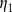, 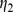, 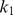, 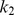, 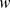. We generated eight simpler models by setting 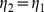, 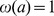, and 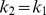.

In the analyses in the section headed ‘Reaction times during habit execution’, we assumed that reaction times in the second stage are inversely related to the probability of executing an action sequence in the first stage. As such, if subject has taken action 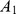 in the first stage, and action 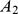 in the second stage, then model prediction of the reaction time of 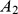 will be:
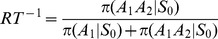
(14)For the second analysis in the section headed ‘Behavioral Modeling: Bayesian model selection’, we aimed to remove the effect of action sequences in the second stage choices. We used eight models same as above, but the probability that the model assigns to action *a* in state 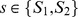, was defined as:
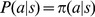
(15)Which indicates probability of taking each action in each slot machine is guided only by the rewards earned on that slot machine, and not by the action sequences in the first stage.

#### Model selection

Since the two families of models that we are comparing are not nested in each other, we can't use classical model selection. Instead, we use a Bayesian model selection for comparing these two families of models [31]. We first calculated the model evidence for each model using the Laplace approximation [59], [60], and then calculated the exceedance probability favoring each family, (taking model identity as a random effect) using the ‘spm_compare_families’ routine in the spm8 software. Within each family, exceedance probabilities were calculated using the ‘spm_BMS’ routine [32].

The Laplace approximation requires a prior assumption of probability distributions over the free parameters of models. Similar to the previous study [23], for parameters between zero and one (learning rates, reinforcement eligibility, weight parameter), we assumed a Beta(1.1, 1.1) distribution; for exploration-exploitation parameters we assumed a Gamma(1.2, 5) distribution, and for perseveration parameters, a Normal(0, 1) distribution was assumed. The Laplace approximation includes finding the maximum a posteriori (MAP) parameter estimates. For this purpose, we used the IPOPT software package [61] for nonlinear optimization, and the DerApproximator package [62] in order to estimate the Hessian at the MAP point.
